# Longitudinal Assessments of Normal and Perilesional Tissues in Focal Brain Ischemia and Partial Optic Nerve Injury with Manganese-enhanced MRI

**DOI:** 10.1038/srep43124

**Published:** 2017-02-23

**Authors:** Kevin C. Chan, Iris Y. Zhou, Stanley S. Liu, Yolandi van der Merwe, Shu-Juan Fan, Victor K. Hung, Sookja K. Chung, Wu-tian Wu, Kwok-fai So, Ed X. Wu

**Affiliations:** 1 NeuroImaging Laboratory, University of Pittsburgh, Pittsburgh, Pennsylvania, United States; 2 UPMC Eye Center, Eye and Ear Institute, Ophthalmology and Visual Science Research Center, Department of Ophthalmology, University of Pittsburgh, Pittsburgh, Pennsylvania, United States; 3 Department of Bioengineering, Swanson School of Engineering, University of Pittsburgh, Pittsburgh, Pennsylvania, United States; 4 Center for the Neural Basis of Cognition, University of Pittsburgh and Carnegie Mellon University, Pittsburgh, Pennsylvania, United States; 5 Louis J. Fox Center for Vision Restoration, University of Pittsburgh, Pittsburgh, Pennsylvania, United States; 6 New York University (NYU) Langone Eye Center, NYU Langone Medical Center, Department of Ophthalmology, NYU School of Medicine, New York, New York, United States; 7 Department of Electrical and Electronic Engineering, The University of Hong Kong, Pokfulam, Hong Kong, China; 8 Athinoula A. Martinos Center for Biomedical Imaging, Department of Radiology, Massachusetts General Hospital and Harvard Medical School, Charlestown, Massachusetts, United States; 9 School of Biomedical Sciences, The University of Hong Kong, Pokfulam, Hong Kong, China; 10 Department of Ophthalmology, The University of Hong Kong, Pokfulam, Hong Kong, China

## Abstract

Although manganese (Mn) can enhance brain tissues for improving magnetic resonance imaging (MRI) assessments, the underlying neural mechanisms of Mn detection remain unclear. In this study, we used Mn-enhanced MRI to test the hypothesis that different Mn entry routes and spatiotemporal Mn distributions can reflect different mechanisms of neural circuitry and neurodegeneration in normal and injured brains. Upon systemic administration, exogenous Mn exhibited varying transport rates and continuous redistribution across healthy rodent brain nuclei over a 2-week timeframe, whereas in rodents following photothrombotic cortical injury, transient middle cerebral artery occlusion, or neonatal hypoxic-ischemic brain injury, Mn preferentially accumulated in perilesional tissues expressing gliosis or oxidative stress within days. Intravitreal Mn administration to healthy rodents not only allowed tracing of primary visual pathways, but also enhanced the hippocampus and medial amygdala within a day, whereas partial transection of the optic nerve led to MRI detection of degrading anterograde Mn transport at the primary injury site and the perilesional tissues secondarily over 6 weeks. Taken together, our results indicate the different Mn transport dynamics across widespread projections in normal and diseased brains. Particularly, perilesional brain tissues may attract abnormal Mn accumulation and gradually reduce anterograde Mn transport via specific Mn entry routes.

Manganese (Mn) has been increasingly used as a positive T1-weighted contrast agent in magnetic resonance imaging (MRI) to study the structures and functions of the central nervous system *in vivo*. Due to the paramagnetic nature of Mn ions and the similar biochemical properties as calcium, Mn has been introduced for neuronal tract tracing, enhancement of neuroarchitecture and functional brain mapping^1^,^2^,^3^,^4^. Mn is also an essential constituent of two important metalloproteins involved in the pathophysiology of the brain. Mn binds to the mitochondrial Mn superoxide dismutase enzyme, which acts against cellular oxidative stress. It can also bind to glutamine synthetase, which is a glial specific enzyme for regulating extracellular glutamate and reducing glutamate excitotoxicity^5^,^6^,^7^,^8^,^9^.

Because of the complex properties of Mn, it remains unclear how Mn is transported dynamically in normal and injured brains, and how Mn accumulates and enhances different brain tissues for MRI detection. A better understanding of the sensitivity and specificity of Mn detection under different experimental conditions can help design optimal tests targeting the normal physiology and pathophysiology of the brain. In this study, we hypothesize that Mn-enhanced MRI can indicate different mechanisms of brain circuitry and neurodegeneration by examining different Mn entry routes and the spatiotemporal Mn distributions across widespread projections in normal and injured brain tissues. We used Mn-enhanced MRI to detect Mn distribution in the normal brains of different rodent species upon systemic or intravitreal Mn injection. In addition, we evaluated the spatiotemporal profiles of Mn enhancement in 3 different types of cerebral ischemic injuries induced by photothrombotic cortical injury (PCI), transient middle cerebral artery occlusion (tMCAO), and neonatal hypoxic-ischemic (HI) injury, and confirmed the alterations in biochemical processes that occurred in the enhanced perilesional tissues using immunohistochemical markers. Lastly, we inspected how partial optic nerve lesions affect primarily and secondarily the anterograde transport of Mn in the injured and uninjured, perilesional visual pathways over time. Our results indicate that Mn-enhanced MRI can be an important *in vivo* technique for dynamically assessing the neural circuitry and degenerative events in localized regions across the brain. In particular, it may help characterize the pathophysiological properties of perilesional brain tissues via different routes of Mn entry.

## Results

### Exogenous Mn enhanced individual brain nuclei at varying rates and peak times after systemic administration to healthy rodents

**Figure 1:**
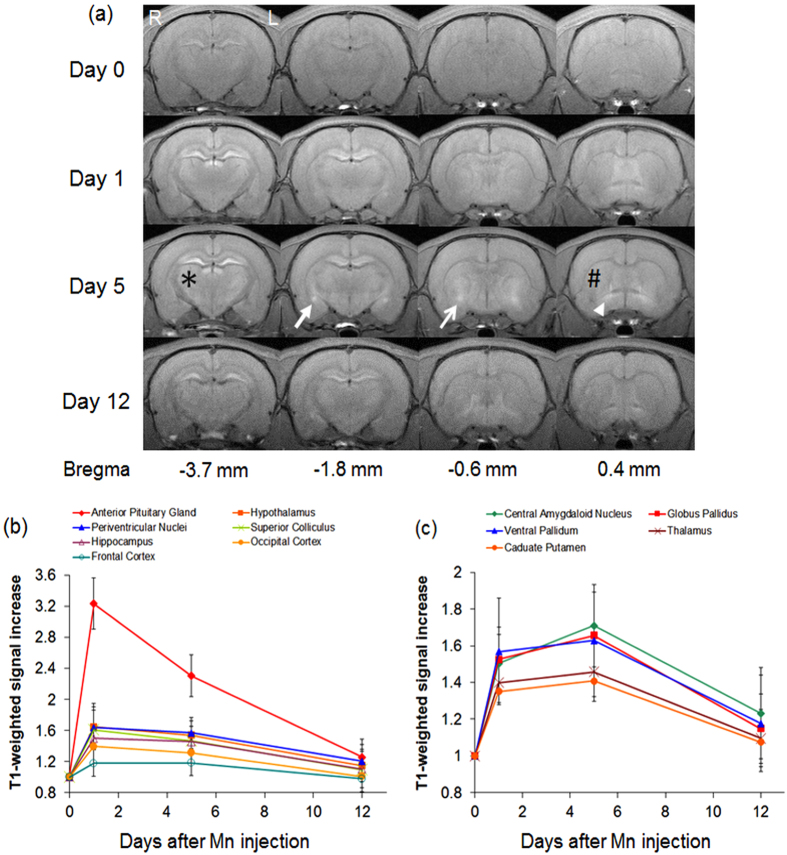
Mn transport dynamics in healthy rat brains upon systemic Mn administration. (**a**) Representative T1-weighted MRI of signal increase at 1, 5 and 12 days after systemic Mn administration relative to pre-injection at Day 0. Note the apparent maximal enhancement at Day 5 in the central amygdaloid nucleus (solid arrow), globus pallidus (open arrow), ventral pallidum (arrowhead), caudate putamen (#), and thalamus (*) when compared to other time points; (**b** and **c**) Time profiles of T1-weighted signal increases in different brain components (Error bars represent ± standard deviation). Significant increases in T1-weighted signal intensities were observed in all measured brain nuclei at 1 day and 5 days after systemic Mn injection relative to the baseline or 12 days after Mn injection (Post-hoc Sidak’s multiple comparisons correction tests, p < 0.05). Note the maximal enhancement at Day 1 in the brain components in (**b**), compared to those with maximal enhancement at Day 5 in (**c**). The anterior pituitary gland and the central amygdaloid nucleus also showed statistically significant differences in T1-weighted signal intensities between Day 1 and Day 5. (Post-hoc Sidak’s multiple comparisons correction tests, p < 0.05).

Upon systemic Mn administration to healthy adult rats (experiment 1), longitudinal T1-weighted MRI showed increased signal intensities to varying extents in both cortical and subcortical brain nuclei at Day 1 and Day 5 compared to pre-injection and Day 12 (Fig. 1). Mn enhancement was the most intense at Day 1 in the anterior pituitary gland, periventricular nuclei, hippocampus, frontal cortex, hypothalamus, superior colliculus and occipital cortex. In the central amygdaloid nucleus, globus pallidus, ventral pallidum, caudate putamen and thalamus, Mn enhancement was apparently more intense at Day 5 than the other 3 time points measured.

### Mn preferentially accumulated in perilesional tissues expressing gliosis or oxidative stress after photothrombotic cortical injury, transient middle cerebral artery occlusion, and neonatal hypoxic-ischemic brain injury

**Figure 2:**
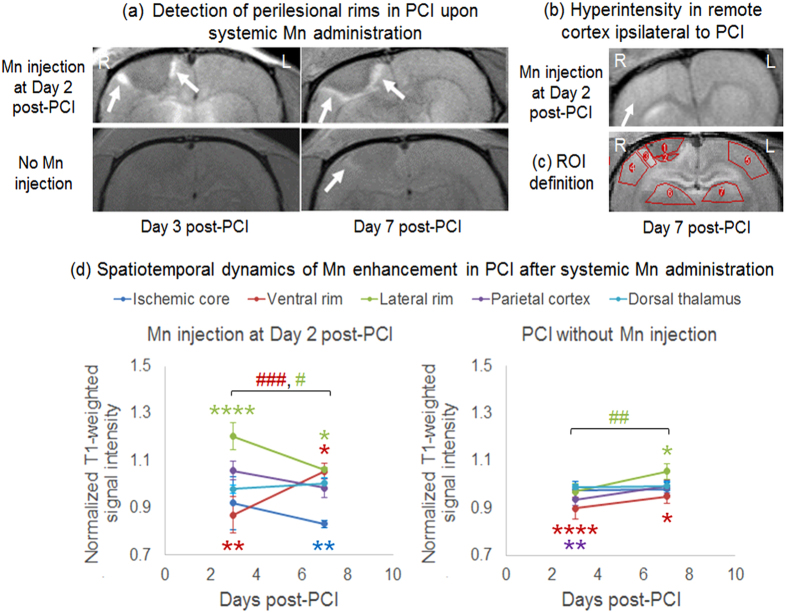
Mn-enhanced MRI of photothrombotic cortical injury (PCI). (**a**) T1-weighted MRI at the level of the hypointense ischemic core at 3 and 7 days after PCI, showing hyperintensity in the perilesional rims (arrows) after systemic Mn injection at Day 2 (top row). Mild hyperintensity was also observed in the perilesional rim (arrow) at 7 days after PCI without Mn injection (bottom row); (**b**) T1-weighted MRI at about 2 mm anterior to the ischemic core at 7 days after PCI, showing hyperintensity in the ipsilesional cortex (arrow) after systemic Mn injection at Day 2; (**c**) Definitions of the regions of interest (ROI) for quantification of brain signal intensities in (**d**); (**d**) Quantitative comparisons of T1-weighted signal intensities in different brain regions at 3 and 7 days after PCI with (left) and without (right) systemic Mn administration at Day 2 (Error bars represent ± standard deviation). The signal intensities were obtained from the left hemisphere and normalized to those in the contralateral right hemisphere. (Post-hoc Sidak’s multiple comparisons correction tests between left and right hemispheres, *p < 0.05; **p < 0.01; ****p < 0.0001; across time, ^#^p < 0.05; ^##^p < 0.01; ^###^p < 0.001).

**Figure 3:**
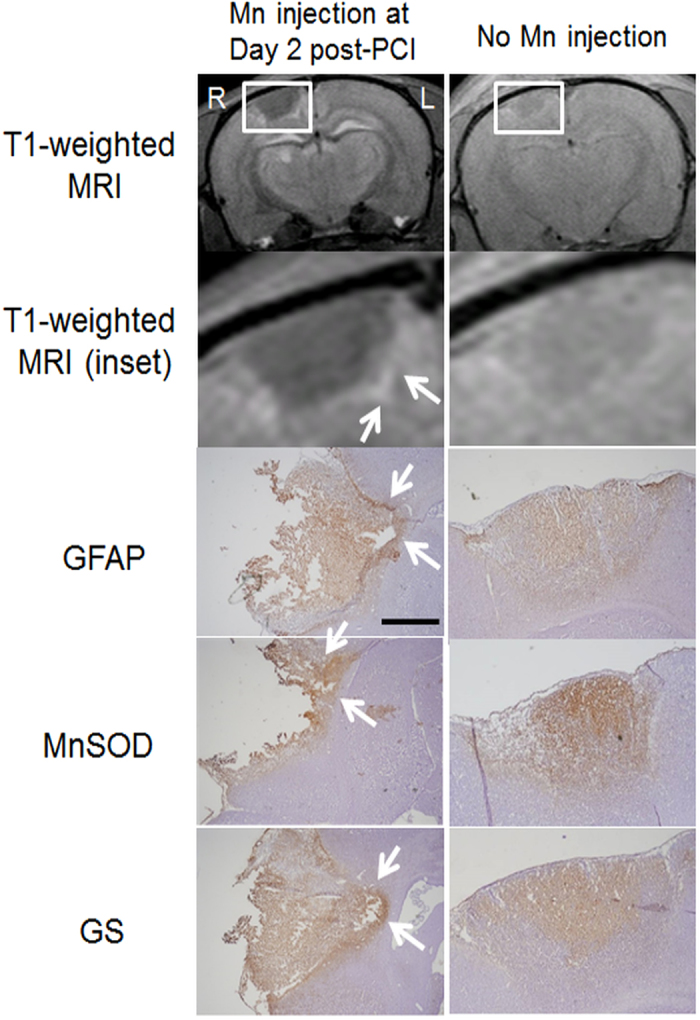
Mn-enhanced MRI and immunohistochemistry of the ischemic core and perilesional cortex after photothrombotic cortical injury (PCI) to the right motor cortex. T1-weighted MRI (top 2 rows) and immunohistochemical stains (bottom 3 rows; counterstained with hematoxylin) at 7 days after PCI with (left column) or without Mn injection at Day 2 (right column). Note the hyperintensities at the perilesional rims (arrows) in both MRI and immunohistochemistry. Magnification for immunohistochemistry = ×4. Scale bar = 0.5 mm. (GFAP: glial fibrillary acidic protein; MnSOD: manganese superoxide dismutase; GS: glutamine synthetase).

Upon systemic Mn administration at 2 days after PCI to the right motor cortex (experiment 2a), T1-weighted hyperintensity was observed in the perilesional rim surrounding the hypointense ischemic core in all animals at Day 3 and Day 7 (Fig. 2a and d). T1-weighted hyperintensity was also observed in the ipsilesional cortex remote to the ischemic core, and occasionally in the subcortical regions after systemic Mn administration to the PCI model (Fig. 2b). When no exogenous Mn was applied to the PCI model, T1-weighted signal enhancement was not apparent in the perilesional rim at 3 days after PCI. However, mild T1-weighted signal enhancement was observed in the perilesional rim at 7 days after PCI without systemic Mn administration (Fig. 2a and d). The T1-weighted signal enhancement at 7 days after PCI colocalized with glial fibrillary acid protein, Mn superoxide dismutase or glutamine synthetase immunoreactivities in the perilesional rim both with and without systemic Mn administration (Fig. 3).

**Figure 4:**
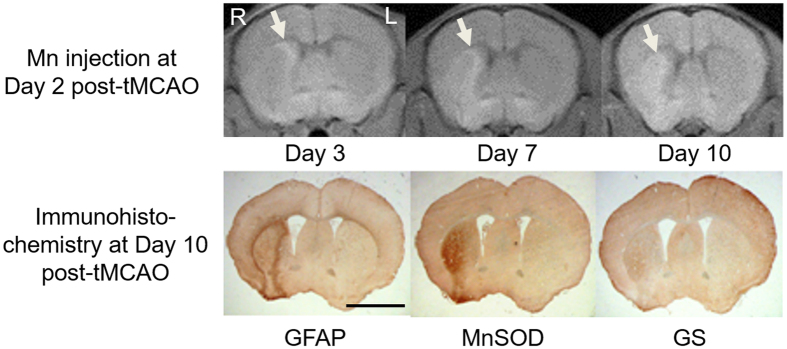
Mn-enhanced MRI and immunohistochemistry of the striatum following 30 min of transient middle cerebral artery occlusion (tMCAO) to the right hemisphere. (Top row) T1-weighted MRI at the level of the striatum at 3, 7 and 10 days following tMCAO. Systemic Mn administration was applied at 2 days after tMCAO; (Bottom row) Immunohistochemical staining of glial fibrillary acidic protein (GFAP), manganese superoxide dismutase (MnSOD) and glutamine synthetase (GS) at 10 days after tMCAO. Magnification = ×4. Scale bar = 3 mm.

Upon systemic Mn administration at 2 days after unilateral tMCAO (experiment 2b), a significant increase in T1-weighted signal intensity was observed in the perilesional rim compared to the ischemic core at Day 3 (paired t-test, p < 0.05) similar to the PCI model, while the Mn enhancement at 10 days after tMCAO was in good colocalization with glial fibrillary acid protein, Mn superoxide dismutase and glutamine synthetase immunostainings (Fig. 4).

**Figure 5:**
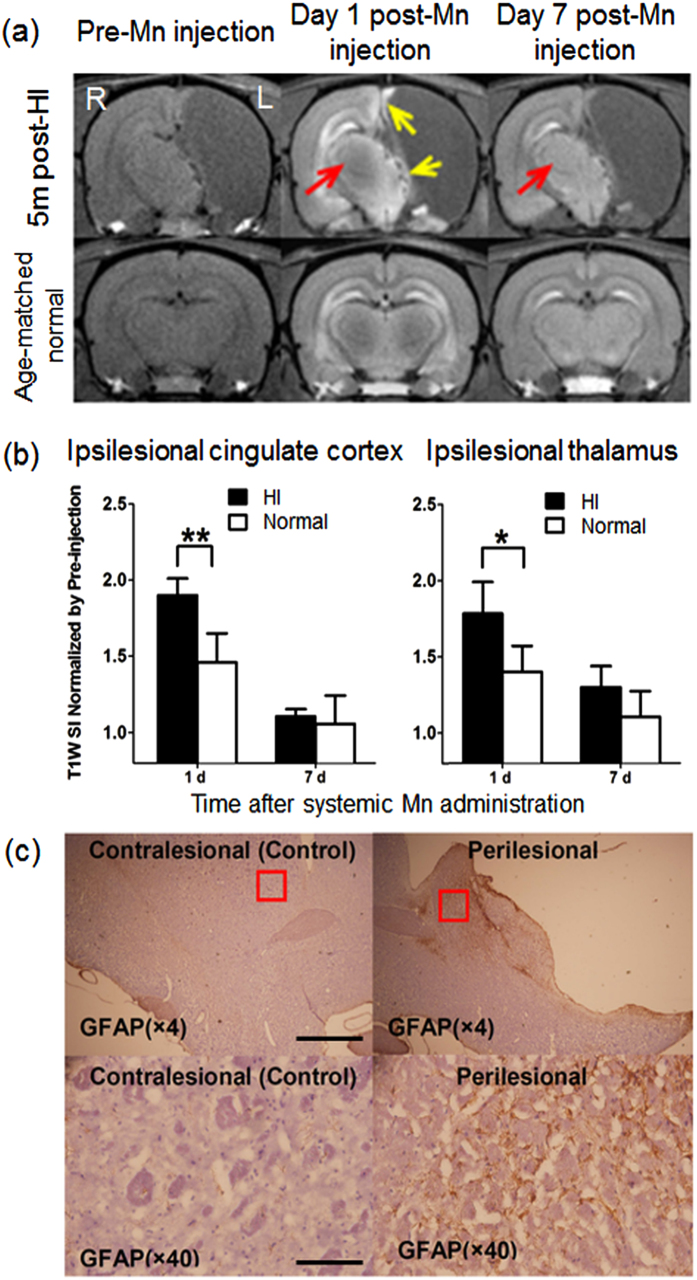
Mn-enhanced MRI and immunohistochemistry of neonatal hypoxic-ischemic (HI) brain injury to the left hemisphere. (**a**) T1-weighted MRI of HI-injured (top row) and age-matched healthy control (bottom row) brains before and at 1 and 7 days after systemic Mn administration and at 5 months after neonatal HI brain injury. In the HI-injured brain, the perilesional rim exhibited Mn-induced signal intensity (SI) increase at Day 1 (yellow arrows), whereas the contralesional thalamus demonstrated a delayed enhancement (red arrows) at Day 7; (**b**) Quantitative comparisons of T1-weighted SI increases in the perilesional cingulate cortex (left) and thalamus (right) of the HI-injured brains and the healthy controls after systemic Mn administration. (Unpaired t-tests between HI-injured and healthy control brains, *p < 0.05; **p < 0.01) (Error bars represent ± standard deviation); (**c**) Immunohistochemical staining of glial fibrillary acidic protein (GFAP, brown) and hematoxylin and eosin (H&E, purple) at ×4 (top row) and ×40 (bottom row) magnification, showing astrocytic hypertrophy in the perilesional region (right column) of the HI-injured group compared to the contralateral hemisphere (left column). Scale bar = 1 mm (×4); 0.1 mm (×40).

Upon systemic Mn administration at 5 months after unilateral neonatal HI brain injury (experiment 2c), T1-weighted hyperintensity was observed in the perilesional regions of the ipsilesional hemisphere, especially in the remaining thalamus and cingulate cortex (Fig. 5a). Neither the contralesional hemisphere nor the age-matched normal control brain exhibited the same extent of Mn contrast enhancement in the homologous areas. The Mn-induced signal changes in the perilesional areas were quantified and validated statistically between the HI-injured group and age-matched normal control group (Fig. 5b). Furthermore, the Mn enhancement in the perilesional tissues colocalized with immunohistochemical expression of glial fibrillary acid protein staining (Fig. 5c).

### Intravitreal Mn administration to healthy rodents enhanced both visual and non-visual pathways across species

**Figure 6:**
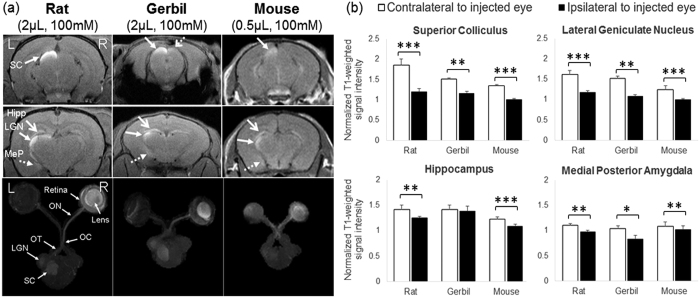
Mn-enhanced MRI of visual and non-visual pathways in the healthy rodent brains upon monocular intravitreal Mn administration. (**a**) Coronal 2D T1-weighted MRI of the rat (left column), gerbil (middle column) and mouse (right column) brains at the levels of the superior colliculus (SC) (top row) and lateral geniculate nucleus (LGN) (middle row) at 1 day after intravitreal Mn injection into the right eye. (Hipp: hippocampus; MeP: posterior medial amygdala). Bottom row shows the axial maximum intensity projection (MIP) of 3D T1-weighted MRI in the primary visual pathways of the same rodent species from the retina to the subcortex. (ON: optic nerve; OC: optic chiasm; OT: optic tract); (**b**) Quantitative assessments of Mn enhancement in the visual (top row) and non-visual (bottom row) brain nuclei at 1 day after intravitreal Mn injection into the right eye. T1-weighted signal intensities of the brain nuclei were normalized to the surrounding muscles and compared between left and right hemispheres of the same species. (Paired t-tests, *p < 0.05; **p < 0.01; ***p < 0.001) (Error bars represent ± standard deviation).

At 1 day after intravitreal Mn injection to the right eyes of healthy adult rats, gerbils and mice (experiment 3), Mn enhancement was observed along the primary visual pathway in the right retina, right lens and right optic nerve, and in the left optic tract, left lateral geniculate nucleus and the superficial layers of the left superior colliculus in all species (closed solid arrows in Fig. 6a). Mild Mn enhancement could also be found in the ipsilateral right superior colliculus (dashed open arrows in Fig. 6a) in gerbils but not rats or mice. In addition, Mn enhancement was observed in the non-visual brain nuclei of the left hemisphere including the posterior medial amygdala (dashed arrows in Fig. 6a) and hippocampus (open solid arrows in Fig. 6a) across species. Quantitative analyses in Fig. 6b verified the significant T1-weighted signal increases in both visual and non-visual brain nuclei of the contralateral hemisphere after monocular intravitreal Mn injection across species.

### Partial optic nerve transection led to gradual loss of anterograde Mn transport along perilesional visual pathway

**Figure 7:**
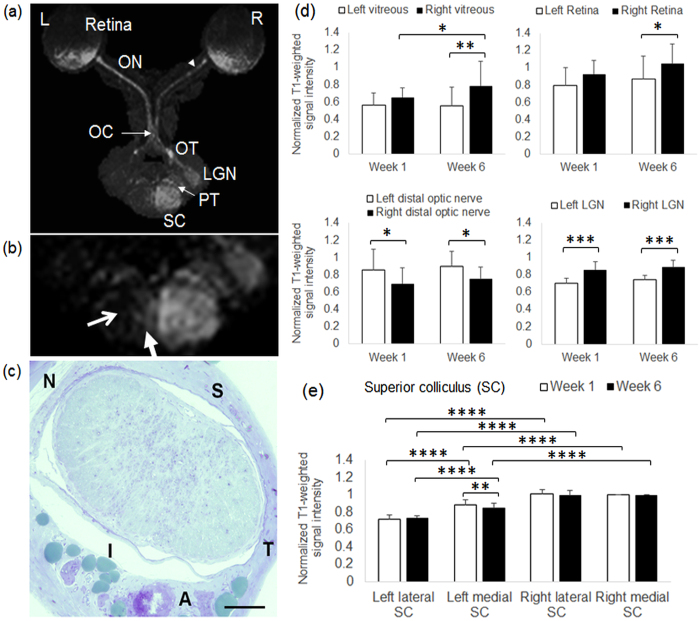
Mn-enhanced MRI of the visual pathway following partial optic nerve injury and binocular intravitreal Mn administration. (**a**) Axial maximum intensity projection (MIP) of 3D T1-weighted MRI of the visual pathway at 6 weeks after partial transection of the right superior intraorbital optic nerve (arrowhead) and 1 day after intravitreal Mn injection to both eyes. (ON: optic nerve; OC: optic chiasm; OT: optic tract; LGN: lateral geniculate nucleus; PT: pretectum; SC: superior colliculus); (**b**) Enlarged MIP image at the level of the superior colliculus. Note the strong hyperintensity in the right superior colliculus projected from the left intact retina and optic nerve, in comparison to the absence of Mn enhancement in the left lateral superior colliculus (open arrow) and the mild hyperintensity in the left medial superior colliculus (solid arrow) projected from the right injured optic nerve; (**c**) Light micrograph of the intraorbital optic nerve section at about 1.5 mm behind the right eye at 6 weeks after partial transection of the superior optic nerve (toluidine blue stain; ×20 magnification; Scale bar = 0.1 mm) (S: superior; I: inferior; T: temporal; N: nasal; A: ophthalmic artery); (**d**) Quantitative assessments of Mn enhancement in the vitreous, retina, the optic nerve distal to the partial transection site, and the lateral geniculate nucleus of both hemispheres at 1 week and 6 weeks after partial optic nerve transection and 1 day after intravitreal Mn injection. T1-weighted signal intensities were normalized to the intensity in the right medial superior colliculus (Post-hoc Sidak’s multiple comparisons correction tests, *p < 0.05; **p < 0.01; ***p < 0.001); (**e**) Quantitative assessments of Mn enhancement in the left lateral and medial superior colliculus, and right medial and lateral superior colliculus at 1 week and 6 weeks after partial optic nerve transection and 1 day after intravitreal Mn injection. T1-weighted signal intensities were normalized to the intensity in the right medial superior colliculus. (Post-hoc Sidak’s multiple comparisons correction tests, **p < 0.01; ****p < 0.0001) (Error bars represent ± standard deviation).

In rats that had received partial transection of the right intraorbital optic nerve for 6 weeks and intravitreal Mn injection into both eyes for 1 day (experiment 4), the right vitreous and retina showed significantly higher T1-weighted signal intensities than the left eye. Distal to the transection site, the right optic nerve and the left lateral geniculate nucleus showed significantly lower T1-weighted signal intensities than the contralateral hemispheres (Fig. 7a and d). In the midbrain, localized T1-weighted hypointensity was found in the lateral half of the left superior colliculus (open arrows in Fig. 7b) at 1 week and 6 weeks after partial transection to the superior region of the right optic nerve. The lateral half of the left superior colliculus had 28 ± 5% and 27 ± 3% lower T1-weighted signal intensities than the right superior colliculus at Week 1 and Week 6 respectively. The medial half of the left superior colliculus also had lower T1-weighted signal intensities than the right superior colliculus but to a smaller extent by 11 ± 6% and 16 ± 5% at Week 1 and Week 6 respectively (Fig. 7e). The T1-weighted signal intensity of the left medial superior colliculus was significantly higher at Week 1 than Week 6, and was significantly higher than that of the left lateral superior colliculus at both time points. No statistical significance was found when comparing between the lateral and medial halves of the right superior colliculus at the same time points, or between Week 1 and Week 6 in the left lateral superior colliculus or the right superior colliculus (Fig. 7e).

## Discussion

Although previous Mn-enhanced MRI studies in healthy rodents showed that systemic Mn administration can enhance most brain tissues within a day^10^,^11^, the specific cellular fate of Mn across the brain following overexposure remain incompletely understood^11^,^12^,^13^. In experiment 1, the varying rates and peak times of Mn enhancement in individual brain nuclei may be explained partly by the differences in baseline metalloprotein levels, brain connectivity and brain activities. After systemic administration, Mn can transport from the plasma into the brain parenchyma across the cerebrospinal fluid and cerebral capillaries^14^. Mn can also diffuse into the brain and enter excitable cells via voltage-gated calcium channels^10^,^11^. The brain structures in Fig. 1b with maximal Mn enhancement at Day 1 are known to possess high contents of unsaturated glutamine synthetase^14^,^15^ as well as high baseline glutaminergic activities^16^, both of which may take up the diffused Mn leading to T1-weighted hyperintensity. In contrast, in brain regions with low glutamine synthetase contents such as the striatum, T1-weighted signal enhancement appeared less pronounced at Day 1. At Day 5, Mn enhancement persisted in the brain structures in Fig. 1b, indicative of the slow efflux of Mn within the first week of systemic Mn administration^17^. On the other hand, exogenous Mn appeared to accumulate further at Day 5 in the central amygdaloid nucleus, globus pallidus, ventral pallidum, caudate putamen and thalamus. While there are many parts of the neocortex innervating the caudate putamen, the caudate putamen provides efferent fibers to ventral pallidum and globus pallidus, which may allow continual Mn transport between subcortical structures. The amygdaloid complex and thalamus are also reciprocally connected with multiple cortical sensory systems^14^. Our results in Fig. 1c suggest that Mn is redistributed through axonal transport in the late phase of systemic Mn administration. Taken together, the Mn-enhanced MRI observations here extend beyond previous findings focusing on the early phase of systemic Mn administration, and demonstrated the intrinsic properties of systemic Mn uptake and continuous redistribution across normal neuronal pathways before global clearance from the brain within a 2-week timeframe. This may provide a basis for biophysical modeling and prediction of Mn transport dynamics across the brain. It has been shown that Mn accumulation in mitochondria may inhibit calcium efflux in the brain^18^,^19^. However, it remains unclear whether there is any distribution correlation between Mn and calcium, and how their distributions are influenced by each other^20^,^21^,^22^,^23^. Future directions are envisioned that examine the overall distributions of both intracellular and extracellular metal ions upon administration of other metal ions.

After acute stroke, the rescued penumbra may be affected by selective neuronal loss and glial activation, which may hinder functional recovery^24^. To date, limited *in vivo* techniques are available to delineate the penumbra regions accurately for better monitoring and targeted intervention of post-stroke events. In experiment 2, when Mn superoxide dismutase activity transiently peaked in the peri-infarct areas at about 7 days after PCI^25^, mild T1-weighted signal enhancement was also observed at the perilesional rim even without systemic Mn administration, suggestive of delayed upregulation of endogenous Mn^26^,^27^. In contrast, after exogenous Mn injection to PCI, tMCAO and HI-injured models, we demonstrated a stronger and earlier detection of Mn enhancement in the perilesional tissues than endogenous Mn upregulation. While the origins of Mn enhancement in neurodegeneration have been debatable in the past years^28^,^29^,^30^,^31^, our experiments indicated the good colocalization of Mn enhancement patterns with glial fibrillary acid protein, Mn superoxide dismutase or glutamine synthetase stains consistently across different brain ischemia models. In the perilesional tissues, astroglial cells that are highly immunostained for glial fibrillary acid protein would secrete reactive oxygen species, resulting in an upregulation of Mn superoxide dismutase and glutamine synthetase which are Mn dependent enzymes^32^. In addition, glutamate could be released in the peri-infarct areas by increasing the activation of neuronal glutamate receptors, which in turn increased membrane permeability to calcium in astrocytes^7^,^33^,^34^. As a calcium analog^35^, it is possible that exogenous Mn may enhance MRI detection of gliosis and oxidative stress. In summary, our experimental findings support the hypothesis that Mn is continuously redistributed differently between normal and injured brains after systemic administration to healthy rodents and brain ischemia rodent models. Our Mn-enhanced MRI observations may help better understand the mechanisms related to the uptake, distribution and action of Mn under different types of brain ischemia. Given that Mn superoxide dismutase and glutamine synthetase activities may increase upon Mn administration to normal and ischemic animals^36^,^37^,^38^, whereby Mn may trigger the scavenging of superoxide and hydroxyl radicals^36^, it is also possible that systemic Mn administration at low doses could exert antioxidative effects to some extents and preserve brain tissues at remote sites from delayed secondary damage (see Supplementary Figs S1 and S2). Such combined Mn administration and MRI detection approaches may be useful for investigation of post-injury cellular events and functional reorganization.

Apart from systemic Mn administration, local Mn administration may provide an excellent imaging model system for understanding the mechanisms of fiber formation, degeneration and plasticity in specific systems of the brain^35^,^39^,^40^,^41^,^42^,^43^,^44^. Within the visual system, there is increasing evidence that suggest the role of the retinal ganglion cells for both visual and non-visual functions^45^. However, the sensitivity of Mn-enhanced MRI for tracing retinal projections to different brain nuclei has not yet been fully explored. It is either incompletely understood how Mn enters, transports and accumulates within the brain circuitry after local administration^35^,^46^,^47^. Upon monocular intravitreal Mn injection to rats, gerbils, and mice, we demonstrated in experiment 3 the feasibility of submillimeter resolution Mn-enhanced MRI for *in vivo* examination of the retinal projections to both visual and non-visual brain nuclei. Apart from Mn enhancement of the primary visual centers along the retinocollicular and retinogeniculate pathways, a consistent unilateral enhancement in the posterior medial amygdala and hippocampus was observed in the contralateral hemisphere across rodent species. This may be partly ascribed to the direct communication between melanopsin-expressing retinal ganglion cells and the medial amygdala^45^,^48^,^49^,^50^, the existence of the retinolimbic pathway between the retina and the hippocampus^51^, or other non-specific enhancements^52^. Understanding the retinal inputs to both visual and non-visual brain nuclei simultaneously may help depict the underlying neural circuit in normal physiology and in diseases that involve widespread brain impairments such as glaucoma^53^,^54^,^55^. Future Mn-enhanced MRI studies are envisioned that measure the widespread retinal projections in normal development, disease, plasticity and therapy in longitudinal studies.

In addition to local Mn tracing of normal neural pathways, we extended beyond our previous cross-sectional 3D Mn-enhanced MRI study^40^, and evaluated longitudinally the primary and secondary degeneration along the retinocollicular projections after partial optic nerve injury in experiment 4. Previous histological work showed that partial transection of the intraorbital optic nerve is accompanied by the spreading damage of secondary degeneration, which results in further loss of neurons and function away from the initial injury^56^,^57^. After partial transection of the right superior optic nerve, the consistent T1-weighted hypointensity in the left lateral superior colliculus than the right superior colliculus at Week 1 and Week 6 reflected the primary loss of topological connections and Mn transport in consistency with our previous study assessed at Week 1 only^40^. The slightly weaker T1-weighted enhancement in the left medial superior colliculus at Week 1 may be partly due to reduced Mn transport upon spreading of oxidative stress through the inferior retina early after partial optic nerve injury^58^, whereas the further reduction in T1-weighted signal enhancement in left medial superior colliculus at Week 6 may indicate secondary loss of retinal ganglion cells and axons projecting through the uninjured, inferior optic nerve^56^,^58^. This further reduction in Mn enhancement at Week 6 was accompanied by more Mn deposition proximal to the transection site in the vitreous and the retina. The current Mn-enhanced MRI results may offer a sensitive tool for longitudinal assessments of secondary changes along initially uninjured neural connections in different neurodegenerative diseases and injuries. Together with Mn-enhanced MRI of perilesional tissues in brain ischemia, careful selection of Mn entry routes may allow examination of specific neurodegenerative events, as well as longitudinal monitoring of the integrity of topological connections in future studies of pharmacologic interventions against secondary degeneration^57^,^59^,^60^.

## Conclusions

Our results indicate the different Mn transport dynamics in the normal and diseased brains that are detectable by Mn-enhanced MRI globally and quantitatively across time. Specifically, Mn may continuously redistribute in normal individual brain nuclei to different extents over a 2-week timeframe after systemic administration, whereas intravitreal Mn administration to healthy rodents may not only allow track-tracing of primary visual pathways, but also enhance non-visual brain nuclei in the hippocampus and medial amygdala consistently across 3 species. Depending on specific Mn entry routes, perilesional brain tissues may attract abnormal Mn accumulation and reduce anterograde Mn transport over time, leading to enhanced MRI detection of neurodegenerative events such as gliosis, oxidative stress and transport deficits after brain ischemia and partial optic nerve injury. Our results on detecting spatiotemporal changes of Mn level in the normal brain tissues may help determine the capacity of Mn uptake in individual brain nuclei, as well as the widespread projections of the brain circuitry. The characterization of Mn enhancement in perilesional tissues may help monitor the pathophysiological properties and evolutionary changes of salvageable tissues, which in turn may guide potential targets for improving functional consequences during primary and secondary neurodegeneration.

## Methods

### Animal Preparation

Four major sets of Mn-enhanced MRI experiments were performed in order to examine the spatiotemporal patterns of Mn enhancement in normal and perilesional tissues of healthy rodents and rodents after focal brain ischemia or partial optic nerve injury. Animals were maintained in an AAALAC-accredited animal facility with a 12 hr light/dark cycle with standard rodent chow and water available ad libitum. All experiments were performed in accordance with the Association of Research for Vision and Ophthalmology Statement for the Use of Animals in Ophthalmic and Vision Research. The protocol was approved by the animal care and use committee of The University of Hong Kong.

### Experiment 1: Mn-enhanced MRI of Mn transport dynamics in normal brain nuclei upon systemic Mn administration

Adult Sprague-Dawley rats (250–300 g, n = 6) were prepared and injected intraperitoneally with MnCl_2_ solution at 45 mg/kg and 100 mM. Mn-enhanced MRI was performed before, and at 1, 5 and 12 days after injection.

### Experiment 2: Mn-enhanced MRI of perilesional rims in brain ischemia upon systemic Mn administration

#### Photothrombotic cortical injury (PCI)

Adult Sprague-Dawley rats (200–250 g, n = 8) were subjected to PCI in the center of the right motor cortex using the Rose Bengal technique^25^, and were divided into 2 groups (n = 4 each). Two days after surgery, one group was administered with an intraperitoneal injection of MnCl_2_ solution (45 mg/kg, 100 mM), while the other group received no Mn injection. T1-weighted MRI was performed to both groups at 3 and 7 days after surgery, whereby two rats from each group were sacrificed for histology after MRI examinations at Day 7.

#### Transient middle cerebral artery occlusion (tMCAO)

Adult C57BL/6 N mice (18–22 g, n = 8) were subjected to intraluminal occlusion of the right middle cerebral artery for 30 min followed by reperfusion under gas anesthesia (induction: 2% halothane in 70% N_2_O/30% O_2_; maintenance: 1% halothane in 70% N_2_O/30% O_2_) at a stabilized body temperature of 37 °C^61^. Two days after surgery, the mice were divided into 2 groups of 4 animals each. One group was administered with an intraperitoneal injection of MnCl_2_ solution (45 mg/kg, 100 mM), while the other group received no Mn injection. T1-weighted MRI was performed at 3, 7, 10, 14 and 21 days after surgery, whereby 2 mice from each group were sacrificed for histology after MRI examinations at Day 10.

#### Neonatal hypoxic-ischemic (HI) brain injury

Apart from PCI and tMCAO, we examined with Mn-enhanced MRI the long-term effects of neonatal HI brain injury on the perilesional tissues. Sprague-Dawley rats (12–16 g, n = 6) underwent unilateral ligation of the left common carotid artery at postnatal day 7 under isoflurane anesthesia, followed by hypoxia in 8% oxygen and 92% nitrogen at 36–37 °C for 2 hours^62^. Another 6 rats were untreated and acted as age-matched healthy control. Five months later, MnCl_2_ solution (60 mg/kg, 100 mM) was slowly infused intraperitoneally at a rate of 15 μl/min for 1 hr. Mn-enhanced MRI was performed before, and at 1 day and 7 days after Mn infusion followed by histology.

### Experiment 3: Mn-enhanced MRI of visual and non-visual pathways in normal brains upon intravitreal Mn administration

Adult Sprague-Dawley rats (226 ± 27 g, n = 6), Mongolian gerbils (66 ± 2 g, n = 4) and C57BL/6 J mice (20 ± 1 g, n = 6) received intravitreal MnCl_2_ injection (100 mM) into the right eye at a volume of 2 μL for rats and gerbils, and 0.5 μL for mice under isoflurane anesthesia. After full recovery, animals returned to their cages, and Mn-enhanced MRI was performed at 1 day after Mn injection. As more than 90% of the rodent retinal ganglion cell axons decussate to the contralateral posterior visual components at the optic chiasm^63^, the Mn enhancement patterns were compared to the brain nuclei of the opposite hemisphere as an internal control.

### Experiment 4: Mn-enhanced MRI of perilesional visual pathway upon partial optic nerve injury

The superior region of the right intraorbital optic nerve of adult Sprague-Dawley rats (200–250 g, n = 10) was partially transected at about 2 mm from the eye using a custom-made thin blade^40^. One week and 6 weeks after surgery, MnCl_2_ solution (3 μL, 50 mM) was injected intravitreally into both eyes of the same animals under isoflurane anesthesia. Animals then recovered and returned to their cages, and Mn-enhanced MRI was performed a day later. Throughout the experiment, the left optic nerve was not transected and the visual pathway projected from the left eye to the right posterior visual brain served as an internal control.

### MRI Protocols

All MRI measurements were acquired utilizing a 7 Tesla Bruker scanner with a maximum gradient of 360 mT/m (70/16 PharmaScan, Bruker Biospin GmbH, Germany). Under anesthesia by inhaled isoflurane (3% induction and 1.5% maintenance), the animals were kept warm under circulating water at 37 °C with respiratory monitoring. Scout T2-weighted images were first acquired in the coronal, transverse and sagittal planes with a rapid-acquisition-with-relaxation-enhancement (RARE) pulse sequence to position the subsequent MR images along standard anatomical orientations in a reproducible manner.

For experiment 1, 2D T1-weighted RARE pulse sequence was employed using a receive-only surface coil with the following parameters: field-of-view (FOV) = 3.2 × 3.2 cm^2^, matrix size = 256 × 256, slice thickness = 1 mm, number of slices = 10, repetition time/echo time (TR/TE) = 400/7.5 ms, RARE factor = 4, number of averages = 16.

For experiment 2, rats with PCI or HI injury were imaged using a receive-only surface coil, whereas mice with tMCAO were imaged using a transmit-receive volume coil. 2D T1-weighted RARE sequences were acquired with TR/TE = 400/7.5 ms, RARE factor = 4, and matrix size = 256 × 256. For the PCI and HI injury models, FOV = 3.2 × 3.2 cm^2^, slice thickness = 1 mm, and number of averages = 16. For the tMCAO model, FOV = 2.0 × 2.0 cm^2^, slice thickness = 0.7 mm, and number of averages = 20.

For experiment 3, a receive-only surface coil was used for imaging rats and gerbils, and a transmit-receive volume coil was used for imaging mice. 2D T1-weighted MRI was acquired in the coronal plane using RARE sequences, with TR/TE = 420/7.5 ms, FOV = 32 × 32 mm^2^ for rats and gerbils, and 20 × 20 mm^2^ for mice, in-plane acquisition resolution = 125 × 125 μm^2^, slice thickness = 0.8 mm, number of slices = 10, RARE factor = 4 and number of averages = 16. In addition, 3D T1-weighted MRI was acquired covering the entire primary visual pathway using the modified driven equilibrium Fourier transform (MDEFT) imaging sequence with inversion time (TI)/TR/TE = 1200/9/3 ms, FOV = 32 × 32 × 11 mm^3^ for rats and gerbils, and 25.6 × 25.6 × 9.6 mm^3^ for mice, acquisition resolution = 200 × 200 × 200 μm^3^, one segment and number of averages = 9.

For experiment 4, all animals were imaged using a receive-only surface coil. 3D T1-weighted MRI was acquired covering the entire primary visual pathway using a RARE sequence, with TR/TE = 250/6.7 ms, FOV = 32 × 32 × 16 mm^3^, acquisition resolution = 200 × 200 × 200 μm^3^, RARE factor = 4 and number of averages = 4.

### Histology

For experiment 2, after MRI examinations at the end time point, a subset of 2 animals from each of the PCI, tMCAO and HI injury groups were transcardially perfused with 4% paraformaldehyde. The brains were then removed, cut into 10 μm sections, and immunostained for GFAP, MnSOD or GS, which are markers for gliosis, oxidative stress and glutamate excitotoxicity, respectively^28^,^38^,^64^. Slices were counterstained with hematoxylin and eosin (H&E) to detect general morphological abnormalities.

For experiment 4, at 6 weeks after partial optic nerve transection, 2 animals were randomly selected after Mn-enhanced MRI for histological verification of the location and severity of optic nerve injury at about 1.5 mm posterior to the eye using toluidine blue staining.

### Data Analysis

For experiment 1, regions of interest (ROI) were manually defined in different brain nuclei of T1-weighted MRI with reference to the rat brain atlas^65^. T1-weighted signal intensities were measured across time using ImageJ v1.44 (Wayne Rasband, NIH, USA), and were normalized to the surrounding saline phantom followed by the pre-injection time point to evaluate the rates of Mn enhancement and clearance in the brain nuclei after systemic Mn administration. The temporal changes of the same brain nuclei were compared using analyses of variance (ANOVA) followed by post-hoc multiple comparisons correction tests via GraphPad Prism v5.00 (GraphPad Software Inc., La Jolla, CA, USA).

For experiment 2, the T1-weighted signal intensities at the ischemic core and the perilesional areas were measured in both PCI and tMCAO models using ImageJ, and were compared with the contralateral hemisphere or across time using ANOVA followed by post-hoc multiple comparisons correction tests. For HI-injured animals, the T1-weighted signal intensities in the remaining tissues of the ipsilesional thalamus and cingulate cortex, and in the contralateral brain tissues were compared with the corresponding locations of age-matched normal controls using two-tailed Student’s t-tests.

For experiment 3, the T1-weighted signal intensities in the superior colliculus, lateral geniculate nucleus, the CA3 region of hippocampus and the posterior medial amygdala of each species were measured in the 2D T1-weighted images using ImageJ with reference to the rodent brain atlases^65^,^66^, and were normalized to the surrounding muscles. The normalized values of the brain nuclei were compared between contralateral hemispheres of the same species using two-tailed Student’s t-tests. In addition, maximum intensity projection was performed onto the 3D T1-weighted images after segmenting the primary visual pathway from the retina to the subcortex in both hemispheres for qualitative evaluation.

For experiment 4, the T1-weighted signal intensities in the vitreous, retina, distal optic nerve, lateral geniculate nucleus, and the medial and lateral regions of the superior colliculus were measured in both hemispheres using ImageJ. These values were normalized to the right medial superior colliculus of each animal, and were compared intra- and inter-hemispherically or between 1 week and 6 weeks after partial optic nerve transection using ANOVA followed by post-hoc multiple comparisons correction tests. Maximum intensity projection was also performed onto the 3D T1-weighted images for qualitative evaluation.

The morphology and intensity of the brain tissues in immunohistochemistry were assessed qualitatively across animal groups in experiments 2 and 4. For experiments 1–4, all quantitative data were presented as mean ± standard deviation. Results were considered statistically significant when p < 0.05.

## Additional Information

**How to cite this article:** Chan, K. C. *et al*. Longitudinal Assessments of Normal and Perilesional Tissues in Focal Brain Ischemia and Partial Optic Nerve Injury with Manganese-enhanced MRI. *Sci. Rep.* **7**, 43124; doi: 10.1038/srep43124 (2017).

**Publisher's note:** Springer Nature remains neutral with regard to jurisdictional claims in published maps and institutional affiliations.

## Supplementary Material

Supplementary Information
