# Supplementary material for: Longitudinal Assessments of Normal and Perilesional Tissues in Focal Brain Ischemia and Partial Optic Nerve Injury with Manganese-enhanced MRI
Source: Sci Rep. 2017 Feb 23;7:43124. doi: 10.1038/srep43124 (PMC5322351; doi:10.1038/srep43124)
Supplement: Supplementary Information [file srep43124-s1.pdf]

SUPPLEMENTARY INFORMATION

Title: Longitudinal Assessments of Normal and Perilesional Tissues in Focal Brain Ischemia and Partial Optic Nerve Injury with Manganese-enhanced MRI

Author list: Kevin C. Chan*, Iris Y. Zhou, Stanley S. Liu, Yolandi van der Merwe, Shu-Juan Fan, Victor K. Hung, Sookja K. Chung, Wu-tian Wu, Kwok-fai So, Ed X. Wu

*Corresponding author

METHODS

In experiment 2b, in order to assess the infarct volume with or without systemic manganese (Mn) administration at 2 days after transient middle cerebral artery occlusion (tMCAO), coronal T2-weighted MRI was performed in addition to T1-weighted MRI to all mice at 3, 7, 10, 14 and 21 days after tMCAO. A rapid-acquisition-with-relaxation-enhancement (RARE) pulse sequence was acquired for T2-weighted MRI under the same dimensions as T1-weighted MRI, with repetition time/echo time = 6500/120 ms, RARE factor = 12, and number of averages = 4. The total brain volumes and the total infarct volumes indicated by T2-weighted hyperintensity were measured in the tMCAO mice using ImageJ v1.44 (Wayne Rasband, NIH, USA). Volumetric values were compared between ipsilesional and contralesional hemispheres using two-tailed Student's t-tests. At 5 months after tMCAO, axial T2-weighted MRI was also performed to the remaining mice to qualitatively assess the brain morphology.

RESULTS

When evaluating the infarct volumes indicated by hyperintensity in T2-weighted MRI, no apparent difference was found in the ipsilesional forebrain between tMCAO models with and without systemic Mn administration (Supplementary Fig. S1a and S1c). In the posterior brain regions remote to the ischemic core, T2-weighted hyperintensity was also observed in the hippocampus and the subcortical structures in all tMCAO animals without systemic Mn administration. However, animals that had received systemic Mn administration at 2 days after tMCAO showed no apparent T2-weighted hyperintensity in the posterior brain at Day 3 or beyond (Supplementary Fig. S1b and S1d). At 5 months after tMCAO, shrinkage of the midbrain and the superior and inferior colliculi was observed in all the 2 remaining animals without systemic Mn administration, but not in animals with systemic Mn administration at 2 days after tMCAO (Supplementary Fig. S2).

DISCUSSION

Upon tMCAO, it has been recognized that brain regions remote to the striatum are less susceptible to oxidative stress[1] and may undergo later onsets for neuronal death due to ischemia and reperfusion[2-4]. While Mn is known to be neurotoxic at high doses, several studies showed the therapeutic potentials of Mn at low doses[5,6]. For example, a Mn complex with a ligand containing oxidizable quinol group demonstrate the potential to concurrently visualize and alleviate oxidative stress[7]. Given that Mn triggered the scavenging of superoxide and hydroxyl radicals6, whereby Mn superoxide dismutase and glutamine synthetase activities may increase upon $MnCl_2$ administration to normal and ischemic animals[6,8,9], it is possible that systemic Mn administration at 2 days after tMCAO could exert antioxidative effects to some extents, and preserve brain tissues

at remote sites from delayed secondary damage. To test this hypothesis, we performed T2-weighted MRI immediately after T1-weighted MRI at each time point to all tMCAO mice, and evaluated the cerebral infarction indicated by T2-weighted hyperintensity[10,11]. We observed a significant reduction in infarct volumes in the posterior brains of the Mn-treated tMCAO mice compared to the untreated tMCAO mice (Supplementary Fig. S1). This effect was further supported by T2-weighted MRI of the remaining unsacrificed animals at 5 months after tMCAO, showing obvious shrinkage of the midbrain without systemic Mn administration but not in animals with Mn administration at 2 days after tMCAO (Supplementary Fig. S2). Recent studies have shown marked neuroprotective effects of Mn complexes against focal ischemic insults up to 6 hours after ischemia[12,13]. The results of this study illustrated the potential neuroprotective effects of Mn after administration at a longer post-ischemic delay. Neither T1- or T2-weighted MRI showed apparent asymmetry in the posterior brain of tMCAO mice after systemic Mn administration at 2 days post-tMCAO. This suggested that Mn accumulation might not be strong enough to induce predominant T2-shortening effects in the ipsilesional posterior brain. No significant difference was observed in the infarct areas in the forebrain of tMCAO mice with and without systemic Mn administration, possibly because the ischemic core was readily damaged before Mn administration.

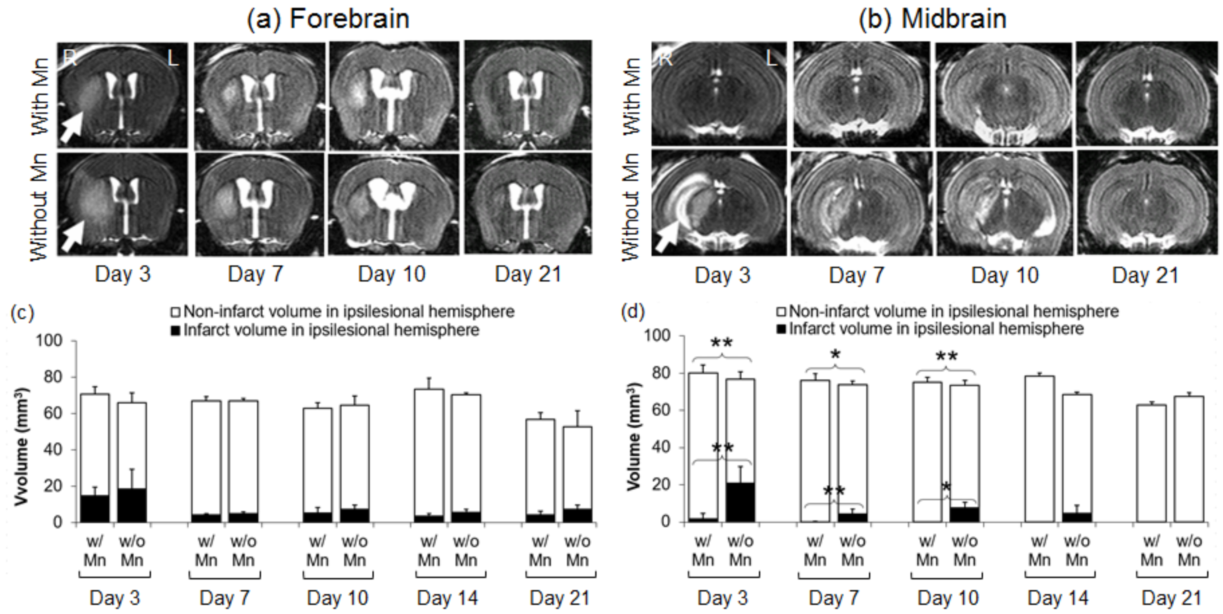

Supplementary Figure S1. Coronal T2-weighted MRI of infarct volumes with and without systemic Mn administration at 2 days after transient middle cerebral artery occlusion (tMCAO) to the right hemisphere. (a and b) T2-weighted MRI of the forebrain **(a)** and midbrain **(b)** at Bregma -0.6 mm and -3.1 mm, respectively, at 3, 7, 10 and 21 days after tMCAO. Note the hyperintensity in the ipsilesional dorsolateral striatum (arrows) in (a) with (top row) and without (bottom row) systemic Mn injection at 2 days after tMCAO. In the midbrain in (b). T2-weighted hyperintensity was also observed in the ipsilesional hippocampus and subcortex (arrow) without Mn injection (bottom row). However, no apparent T2-weighted hyperintensity was observed in the ipsilesional midbrain after systemic Mn injection at 2 days after tMCAO (top row); **(c)** The infarct size in the ipsilesional forebrain indicated by T2-weighted hyperintensity between Bregma -1.7 mm and 1.5 mm was not different between animals with and without systemic Mn injection after tMCAO (unpaired t-tests, $p>0.05$); **(d)** After systemic Mn injection at day 2 after tMCAO, the infarct size in the midbrain between Bregma -5.0 mm and -1.7 mm was significantly reduced compared to animals receiving no Mn injection. [unpaired t-tests of infarct volumes (black) or non-infarct volumes (white) between Mn-injected and uninjected groups, $*p<0.05$; $**p<0.01$] (Error bars represent ± standard deviation)

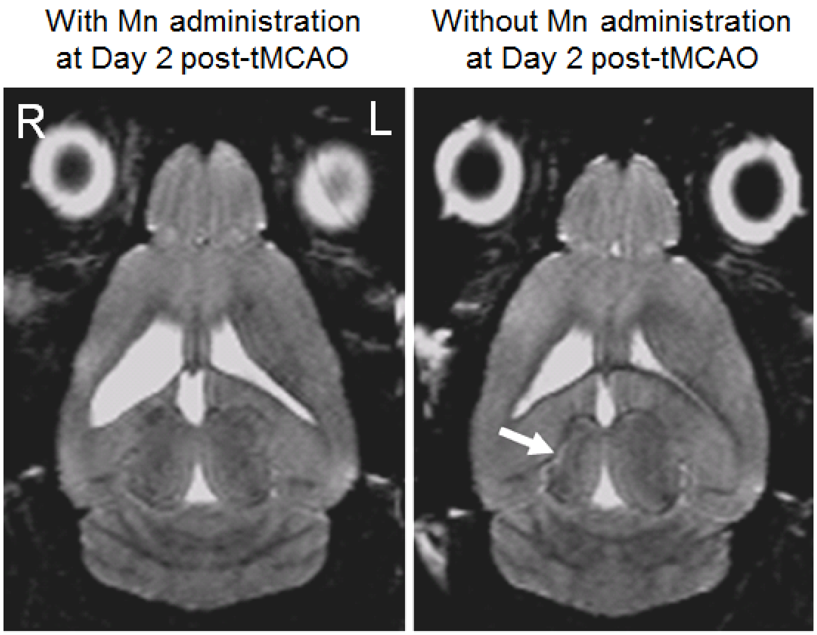

Supplementary Figure S2. Axial T2-weighted MRI of mouse brains at 5 months after transient middle cerebral artery occlusion (tMCAO) with (left) or without (right) systemic Mn administration at 2 days after tMCAO. Apparent shrinkage of the ipsilesional midbrain (arrow) was observed in all mice without Mn administration, but not in the Mn-treated mice.

REFERENCES

1 Homi, H. M., Freitas, J. J., Curi, R., Velasco, I. T. & Junior, B. A. Changes in superoxide dismutase and catalase activities of rat brain regions during early global transient ischemia/reperfusion. *Neurosci Lett* **333**, 37-40 (2002).

2 Pulsinelli, W. A., Brierley, J. B. & Plum, F. Temporal profile of neuronal damage in a model of transient forebrain ischemia. *Ann Neurol* **11**, 491-498, doi:10.1002/ana.410110509 (1982).

3 Gehrmann, J., Banati, R. B., Wiessner, C., Hossmann, K. A. & Kreutzberg, G. W. Reactive microglia in cerebral ischaemia: an early mediator of tissue damage? *Neuropathol Appl Neurobiol* **21**, 277-289 (1995).

4 Maier, C. M. & Chan, P. H. Role of superoxide dismutases in oxidative damage and neurodegenerative disorders. *Neuroscientist* **8**, 323-334 (2002).

5 Song, M., Liu, T., Shi, C., Zhang, X. & Chen, X. Bioconjugated Manganese Dioxide Nanoparticles Enhance Chemotherapy Response by Priming Tumor-Associated Macrophages toward M1-like Phenotype and Attenuating Tumor Hypoxia. *ACS Nano*, doi:10.1021/acsnano.5b06779 (2015).

6 Hussain, S. & Ali, S. F. Manganese scavenges superoxide and hydroxyl radicals: an in vitro study in rats. *Neurosci Lett* **261**, 21-24 (1999).

7 Yu, M. *et al.* A mononuclear manganese(II) complex demonstrates a strategy to simultaneously image and treat oxidative stress. *J Am Chem Soc* **136**, 12836-12839, doi:10.1021/ja507034d (2014).

8 Singh, R. K. *et al.* Potential use of simple manganese salts as antioxidant drugs in horses. *Am J Vet Res* **53**, 1822-1829 (1992).

9 Yang, J. & Wu, E. X. Detection of cortical gray matter lesion in the late phase of mild hypoxic-ischemic injury by manganese-enhanced MRI. *Neuroimage* **39**, 669-679, doi:10.1016/j.neuroimage.2007.09.009 (2008).

10 Ritzl, A. *et al.* Development of brain infarct volume as assessed by magnetic resonance imaging (MRI): follow-up of diffusion-weighted MRI lesions. *J Magn Reson Imaging* **20**, 201-207, doi:10.1002/jmri.20096 (2004).

11 Wang, Y. *et al.* Comparing diffusion-weighted and T2-weighted MR imaging for the quantification of infarct size in a neonatal rat hypoxic-ischemic model at 24h post-injury. *Int J Dev Neurosci* **25**, 1-5, doi:10.1016/j.ijdevneu.2006.12.003 (2007).

12 Mackensen, G. B. *et al.* Neuroprotection from delayed postischemic administration of a metalloporphyrin catalytic antioxidant. *J Neurosci* **21**, 4582-4592 (2001).

13 Vajragupta, O. *et al.* Manganese complexes of curcumin and its derivatives: evaluation for the radical scavenging ability and neuroprotective activity. *Free Radic Biol Med* **35**, 1632-1644 (2003).
